# Recent applications of computational methods to allosteric drug discovery

**DOI:** 10.3389/fmolb.2022.1070328

**Published:** 2023-01-12

**Authors:** Rajiv Gandhi Govindaraj, Sundar Thangapandian, Michael Schauperl, Rajiah Aldrin Denny, David J. Diller

**Affiliations:** ^1^ Computational Chemistry, HotSpot Therapeutics Inc., Boston, MA, United States; ^2^ Medizen Inc., Canton, MA, United States

**Keywords:** allostery, computational modeling, molecular dynamics, structure-based design, fragment-based screening

## Abstract

Interest in exploiting allosteric sites for the development of new therapeutics has grown considerably over the last two decades. The chief driving force behind the interest in allostery for drug discovery stems from the fact that in comparison to orthosteric sites, allosteric sites are less conserved across a protein family, thereby offering greater opportunity for selectivity and ultimately tolerability. While there is significant overlap between structure-based drug design for orthosteric and allosteric sites, allosteric sites offer additional challenges mostly involving the need to better understand protein flexibility and its relationship to protein function. Here we examine the extent to which structure-based drug design is impacting allosteric drug design by highlighting several targets across a variety of target classes.

## Introduction

The majority of naturally occurring biological processes exploit a variety of types of allosteric regulation. This regulation originates in response to various stimuli such as post-translational modifications, point mutations, molecular interactions, and other environmental factors, which ultimately control the specific function of a protein (Nussinov and Tsai, 2013; Lu et al., 2019a; Tee et al., 2021). Knowledge of allosteric effects on proteins comes from over six decades of research since the term “allostery” was first mentioned in 1961 by Monod and Jacob (Monod and Jacob, 1961). Even much before that, in 1904, the notion of the binding of one entity controlling the binding of another at a distinct site was suggested by Bohr (Liu and Nussinov, 2016). No structural explanation was given for this observed phenomenon. Later, Monod and Jacob explained “allosteric inhibition” as a process where an inhibitory molecule binds with no steric hindrance to the substrate. They, along with Changeux, proposed a model in 1965 that described allosteric regulation of an oligomeric system as a symmetrically organized reversibly accessible two state system, where the conformational transitions occur in an all-or-none concerted fashion (Monod et al., 1965; Changeux, 2013). This was immediately debated by an alternative sequential model proposed by Filmer et al., in 1966 that addressed the possibility of differential binding states, which also accounted for negative cooperativity (Koshland et al., 1966). Cooper and Dryden (1984) proposed that protein conformational changes may not be necessary in all cases to regulate distinct sites. Instead, they can be explained using changes in normal mode frequencies and mean-square atomic displacements. At the end of the 20th century, two groups of researchers (Nussinov and coworkers and Ranganathan and coworkers) in parallel advanced the concept of allostery by proposing a conformational ensemble of multiple states of a protein and allosteric communication between protein sectors known as allosteric networks (Lockless and Ranganathan, 1999; Tsai et al., 1999).

In recent groundbreaking work, Hadzipasic et al. (2020) experimentally studied the development of allosteric regulation along evolutionary pathways. Using Aurora A kinase and its allosteric modulator TPX2 along with the calculated ancestral sequence reconstruction, they explained that colocalization of Aurora A and TPX2 is more critical than their coevolution. From the constructed evolutionary pathways, they concluded that the autophosphorylation of the activation loop, which has been present for at least 1 billion years, is the oldest and most conserved mechanism in all Aurora kinases while the regulation by TPX2 *via* protein scaffolding evolved gradually. Also, a gradual increase in the rate of enzymatic function was observed along the evolution of the regulatory partner TPX2. This study also explained that the source of allosteric control is encoded within the kinase rather than in the activation partner with an example of equally observed activation of Aurora A by INCENP, the activation partner of a close homolog Aurora B. The authors ruled out the coevolution of Aurora A and TPX2 by observing an experimentally similar allosteric activation of Aurora A using a generic synthetic substrate and by the fact that none of the TPX2 forms allosterically regulated the precanonical ancestral Aurora A forms even at very high concentrations. Specifically, for proteins controlled by phosphorylation, Pearlman et al. (2011) used a comparative genomics approach to show that nature preferred the evolution of the phosphorylation of serine, threonine and tyrosine residues over the negatively charged aspartate and glutamate residues. Further, a deep mutational scanning study by Leander et al. (2020) suggested the “fold over function” phenomenon, where the critical residues for allosteric control are poorly conserved while the residues critical for the structural stability are highly conserved.

The traditional definition of allostery involves bidirectional communication from a functional site of a protein to a secondary site through conformational changes of the protein. More recently, it has come to also include sites involved in other protein regulation though not necessarily conformationally linked to the orthosteric site. Here we adopt the functional definition proposed by Fenton (2008) in which a ligand is considered an allosteric effector of an orthosteric ligand if three elements are present: first, the allosteric ligand must be chemically distinct from the orthosteric ligand; second, the binding of the allosteric ligand must alter the function of the protein; and third, the allosteric ligand must bind at a non-overlapping site of the protein relative to the orthosteric ligand. This definition of allostery certainly includes the cases where two distinct binding sites are conformationally linked as described in the opening paragraph. It also allows other types of allosteric sites such as scaffolding sites or sites of posttranslational modifications. These sites are often cryptic sites and/or ectosteric sites, where the former is only accessible when a ligand is bound (e.g., as in interleukin-2 (Bowman and Geissler, 2012)) and the latter is a site that does not affect the catalytic site of the protein (e.g., as in cathepsins (Law et al., 2017)) yet still impacts the function of the protein in the context of its cellular pathway.

The pharmaceutical industry has invested heavily in developing drug molecules for various targets which take advantage of allosteric control after multiple allosteric drugs were approved by the FDA such as trametinib and MK2206 that are MEK and AKT inhibitors, respectively. A total of 19 drugs have been approved by the FDA for various diseases that exert their therapeutic effects *via* allosteric mechanisms (Huang et al., 2011). Major advantages of allosteric drugs are manifold including the non-competition to the orthosteric ligand leading to more potency, enhanced selectivity to the specific target resulting in less off-target activity, and better dose requirements. Despite these advantages, treating diseases harnessing allosteric control is not without its own challenges. One such example is assay development which can be significantly more challenging with allosteric sites, particularly those that have no functional impact in biochemical assays.

Allosteric sites can be particularly challenging for computational modeling as well. First, often high-resolution structures of the protein are either not available or lack domains that are critical for the allostery. Even in cases where the structure of the protein is available, many allosteric sites are not evident without a bound ligand. Second, many structure-based design tools rely implicitly on the well-defined shape offered by the relatively deep and rigid pockets found at many orthosteric sites. Shape is often less well defined in allosteric sites. Finally, in most allosteric sites there are no known anchor interactions such as those found from the reaction mechanism of an enzyme or the hinge hydrogen bonds that drive potent binding for ATP competitive protein kinase inhibitors.

### The growing interest in allostery

**FIGURE 1:**
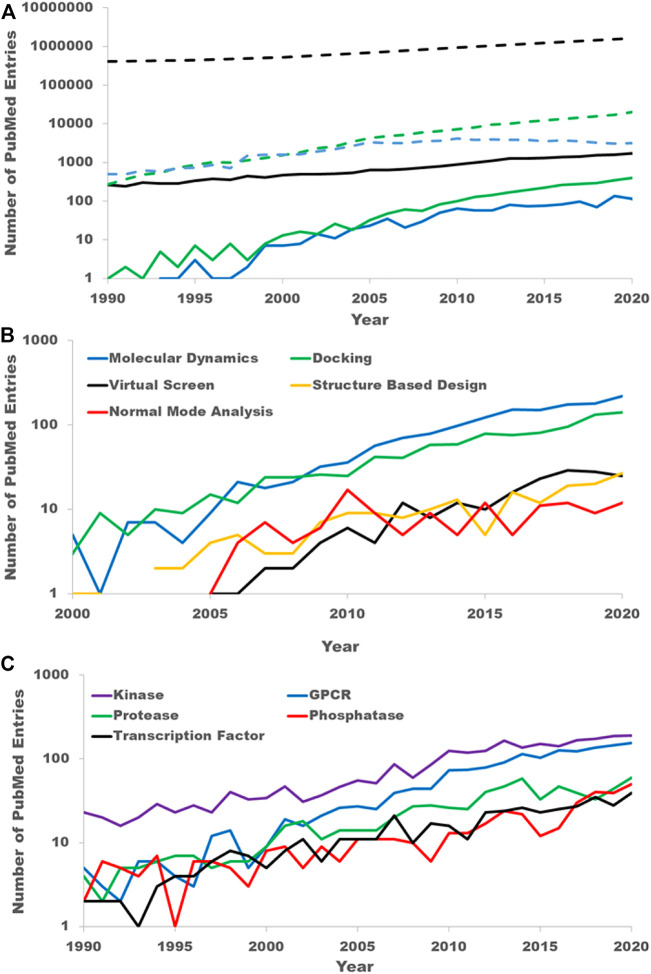
PubMed literature mining. **(A)** The growing interest in allostery as reflected in the scientific literature. The black dashed line is the count of all PubMed entries by year. The blue dashed line is the count of PubMed entries from well-established medicinal chemistry journals. The green dashed line is the count of PubMed entries with clear computational keywords. The solid lines are the counts obtained by further filtering the entries in the dashed line to those containing either keyword “allostery” or “allosteric”. The solid black line is the count of all PubMed entries with either of the keywords “allostery” or “allosteric.” This is referred to as the PubMed allosteric set (PAS) is the text. The solid blue line is a further filtering of the PAS by those entries from the same medicinal chemistry journals. The solid green line is a further filtering of the PAS to those have a clear computational key word. **(B)** Breakdown of the PAS by specific computational techniques. **(C)** Breakdown of the PAS by target family keywords.

To quantify the growing interest in allosteric sites as drug targets, we mined PubMed abstracts. Overall, the number of abstracts in PubMed has grown steadily since 1990 with a doubling time of 14.3 years (Figure 1A). As a first step in analyzing the growth of allostery in the literature, we filtered all PubMed abstracts from 1990 to the present to those that contained one of the keywords “allostery” or “allosteric” in either its title, abstract or keywords. We will refer to this set as the “PubMed allostery set” (PAS). The PAS has grown steadily as well since 1990 with a doubling rate of 10.8 years. In 1990 the PAS accounted for only .06% of publications. While still small overall, this fraction has grown to .1% in 2020.

It is difficult to unilaterally classify publications as drug discovery-oriented simply through keyword searches. To estimate the growth in allosteric sites as drug targets, we filtered the PAS by well-known medicinal chemistry journals: the Journal of Medicinal Chemistry, Bioorganic Medicinal Chemistry, Bioorganic Medicinal Chemistry Letters, the European Journal of Medicinal chemistry, and ACS Medicinal Chemistry Letters. While this certainly will not capture all drug discovery-oriented publications, we believe the growth in these publications reflects the overall growth of drug discovery publications and certainly reflects the growth in interest from the pharmaceutical and biotech companies. Prior to 2000, there were very few publications in these journals that referred to allostery. Since 2000 there has been a significant growth in allostery publications in these journals (Figure 1A). Even with the fewer number of publications the growth rate is near constant from 2005 to 2020 with a doubling rate of 6.6 years with 95% confidence limits of 5.2–8.9 years. Thus, the growth rate in allosteric drug discovery papers appears to far exceed that of the entirety of PubMed or even the PAS.

To quantify the growth in computational studies of allostery, we filtered the PAS by the following keywords: virtual screen, molecular modeling, molecular dynamics (MD), MD simulation, normal mode analysis, elastic network, computational analysis, computational modeling, docking and *in silico*. As with the drug discovery allostery papers, there was little mention of allostery in computational work prior to 2000. Since 2005, the growth in computational allostery publications has a doubling time of 3.6 years with 95% confidence limits of 3.2–4.1 years (Figure 1B). Of the computational allostery publications, half are accounted for with the keyword “molecular dynamics” alone and just over 75% are accounted for by either “molecular dynamics” or “docking.” This is expected as studying allostery in proteins fundamentally requires a protein structure and techniques to understand protein flexibility.

Lastly, we quantified the interest in allosteric sites by protein family. To do this we filtered the PAS with standard keywords for several target families. The counts of abstracts from the PAS for the five most common target families in the PAS, kinases (keyword: “kinase”), GPCRs (keywords: “GPCR” and “G-protein coupled receptor”), proteases (keywords: “protease” and “peptidase”), phosphatases (keyword: “phosphatase”), and transcription factors (keyword: “transcription factor”), are shown in Figure 1C. Kinases have been the most frequently occurring within the PAS, nearly 200 times in 2020, though the gap between kinases and GPCRs has shrunk considerably over the last 15 years. Indeed, the importance of allosteric modulators for both protein kinases (Laufkötter et al., 2022) and GPCRs (Lu and Zhang, 2019; Han et al., 2020; Slosky et al., 2021) drug discovery has been recently reviewed.

The growth in allostery in Pubmed described above is mirrored in a number of different areas relevant to drug discovery. In particular, the allosteric structural database (Fischmann et al., 2009; Cui et al., 2013; Zha et al., 2022) has captured information ranging from known small molecule allosteric modulators to proteins. Consistent with a doubling rate of 10–11 years for Pubmed articles discussing allostery, the number of allosteric modulators, allosteric sites and disease associations in then ASD grew by well over a factor of 2 from 2011 to 2019. The only significant category that saw more modest growth in that time frame was the number of proteins demonstrating allostery.

Due to the increase in interest discussed above and the challenging computational problems, allostery is a new frontier for the development of computational tools. Accordingly, there has been intense effort to develop computational tools to understand allostery (Lu et al., 2019b; Verkhivker, 2021; Ni et al., 2022). The purpose of this review is to assess the extent to which these tools are being used in and impacting ongoing drug discovery programs. In the following sections, we discuss the evolution of small molecule allosteric modulators of several interesting targets from biomedically significant protein families: proteases, phosphatases, nuclear hormone receptors, peptidases and arginine methyltransferases. For each, we highlight the surprising number of different allosteric sites identified across each family and then focus on a single site where allosteric approaches have been extensively pursued, some resulting in success and some in failure. Finally, we will discuss some practical challenges in targeting allosteric sites with a special focus on computational aspects and potential solutions.

### Kinases—Type III allosteric inhibitors of the MEK and WNK families

By avoiding the structurally conserved ATP binding site, allosteric kinase inhibitors have the potential to be highly selective and therefore can be excellent candidates for kinase drug discovery. In large part, this explains why the kinase family appears most frequently in the context of the PAS and in allosteric structures in the PDB.

**FIGURE 2:**
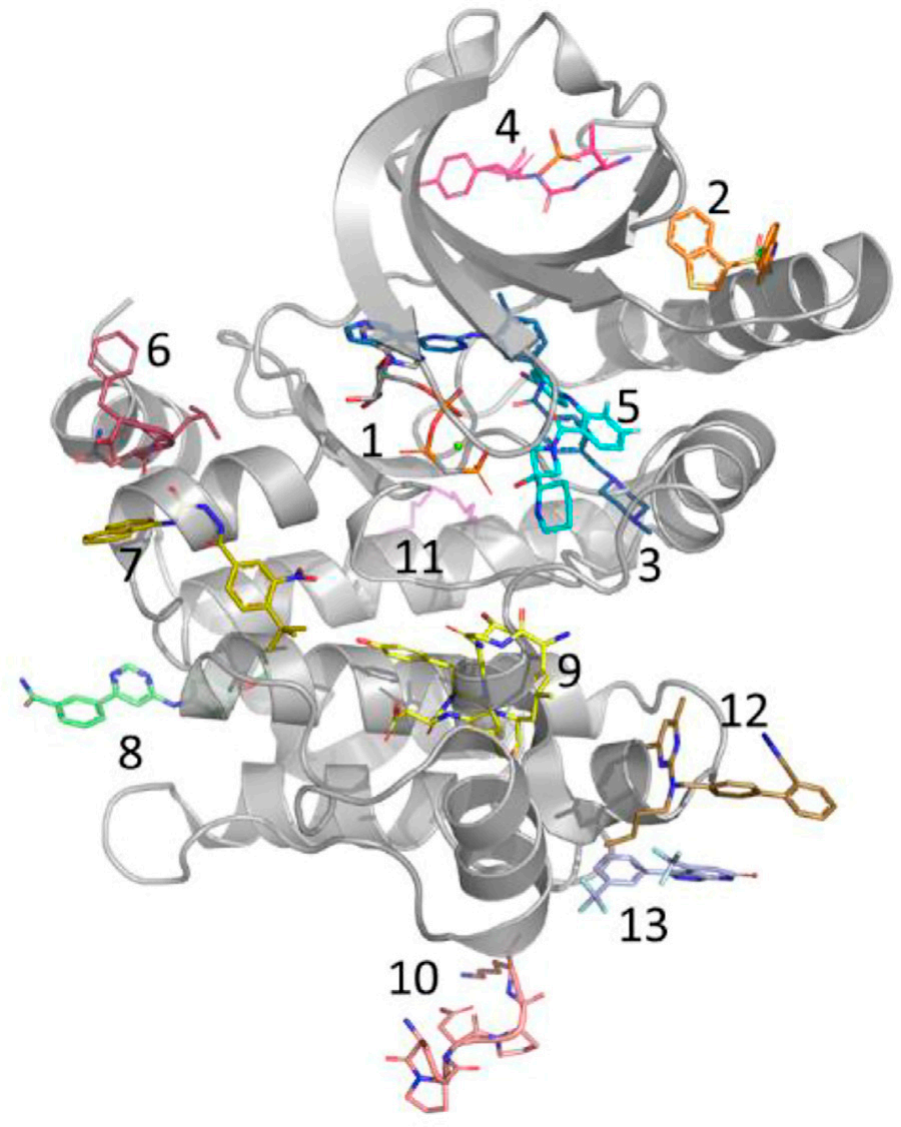
Allosteric Pockets in Kinases. Location of allosteric pockets reported in various Kinase proteins; 1. ATP is not an allosteric site but is included here as a reference (MEK1, 4an2), 2. PIF/HM (PDK1, 4rqk), 3. DFG (C-ABL, 1iep), 4. MPP (MKK4, 3alo), 5. MT3 (MEK1, 4an2), 6. DRS (MAPK8, 1uki), 7. PDIG (Chk1, 3jvs), 8. CMP (c-Abl, 3k5v), 9. AAS (Aurora A, 4c3p), 10. EDI (EGFR, 2rfe), 11. PMP (PKA, 1cmk), 12. DEF (MAPK8, 3o2m), 13. LBP (MAPK14, 3new).

In general, kinase inhibitors have been classified based on their mode of action and location of their binding sites (Figure 2) (Lu et al., 2020). Of those, type I & II inhibitors (Site 1 & 3 of Figure 2) are not considered allosteric, as their binding sites, at least in part, overlap with the orthosteric/ATP site. In contrast, allosteric type III kinase inhibitors bind to a site proximal to the orthosteric/ATP site but with no overlap. Type IV kinase inhibitors include several distinct sites found throughout the kinase domain (Site 2, 4 and 6–13 of Figure 2 **)**. The type III allosteric pocket (Site 5 of Figure 2) has been closely scrutinized due to the approval of 4 drugs that target the MEK family *via* this pocket. While most of the known type III allosteric kinase inhibitors target the MEK family, recently type III inhibitors have been reported for the WNK family as well.

**FIGURE 3:**
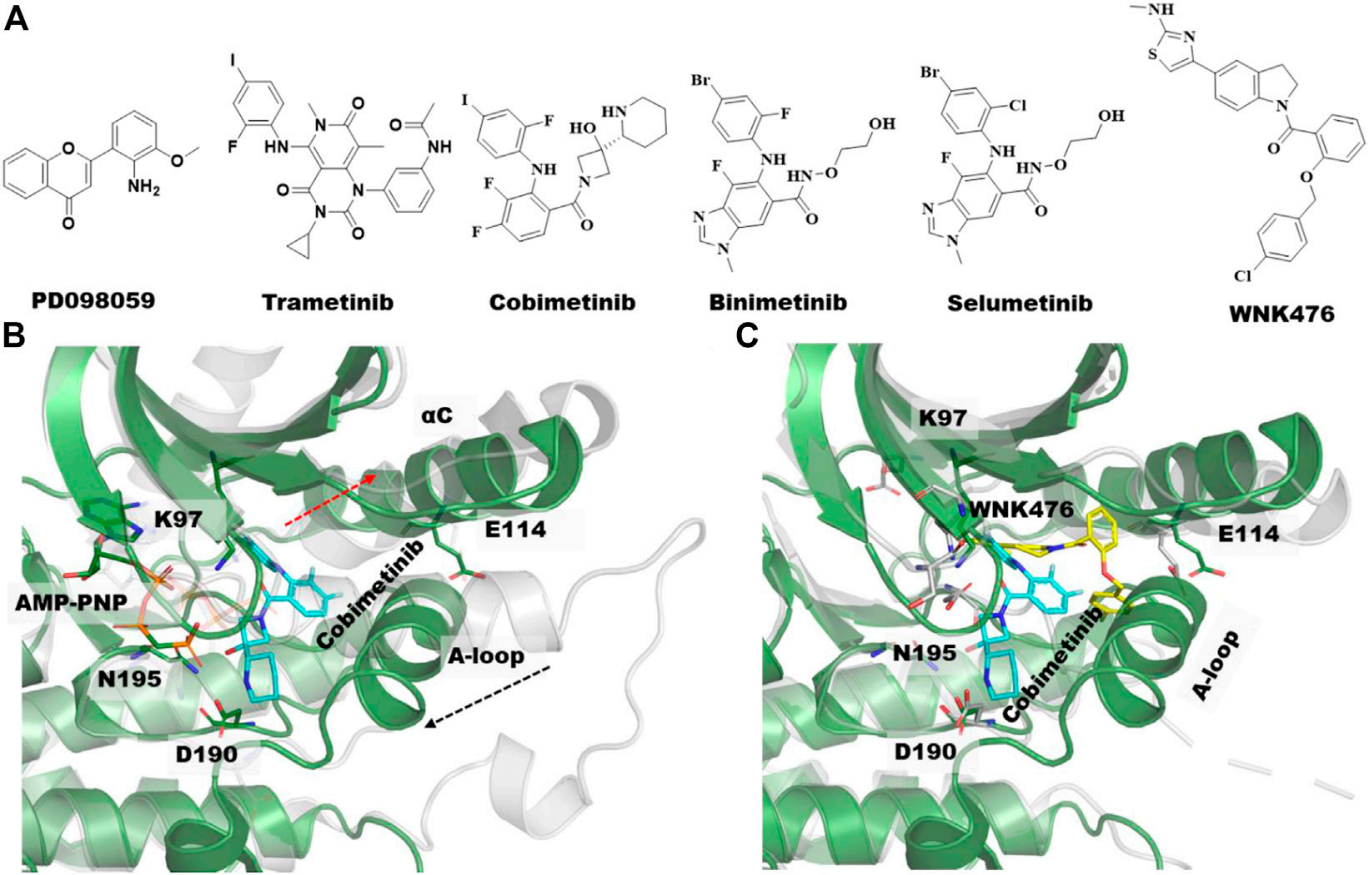
Type III allosteric kinase inhibitors and co-crystal structures. **(A)** Chemical structures of the first identified type III MEK inhibitors along with FDA approved MEK1/2 Inhibitors and WNK476, a type III inhibitor of the WNK kinases. **(B)** Aligned unphosphorylated structure of MEK1 (3w8q, gray transparent cartoon) and MEK1-cobimetinib bound complex structure with AMP-PNP (4an2, green cartoon), the type III inhibitor, Cobimetinib and AMP-PNG is shown in cyan and gray sticks, respectively. Movement of αC-helix and activation loop (A-loop) upon allosteric compound binding are illustrated with dotted red arrow. **(C)** Overlayed crystal structures of MEK1-cobimetinib bound complex (4an2, green cartoon, cyan sticks) and WNK1-WNK476 bound complex (5tf9, gray transparent cartoon, yellow sticks). AMP-PNP is hidden in this complex structure for clarity.

MEK1 is a member of the mitogen-activated protein kinase (MAPK) pathway involved in many key cellular processes including proliferation and differentiation, stress response, and cell death (Robinson and Cobb, 1997). MEK1 and MEK2 are closely related (79% sequence identity), and overactivation of either MEK1 or MEK2 in the MAPK pathway is reported to be responsible for the pathogenesis of inflammation and for nearly 30% of all human cancers (Ohren et al., 2004; Ma and Quirion, 2005). Consequently, MEK1/2 have been studied intensely as drug targets. To date, four MEK inhibitors, including trametinib (IC_50_ = .0007/.0009 µM), binimetinib, selumetinib (IC_50_ = .008 µM), and cobimetinib (IC_50_ = .0009 µM) have been approved by the FDA (Figure 3A) and more than ten inhibitors are in phase I/II trials. Interestingly, none of the four approved drugs are ATP-competitive, rather they are type III kinase inhibitors (Akinleye et al., 2013; Zhao and Adjei, 2014; Cheng and Tian, 2017).

It has been nearly 25 years since the first type III allosteric MEK inhibitor, PD098059, was identified (Figure 3A). This compound was shown to have an allosteric mechanism of action that prevents the activation of MEK by Raf (Alessi et al., 1995). MEK1/2 are regulated and activated by Raf phosphorylation of two serine residues in the activation loop (A-loop), S218 and S222. Since dual phosphorylation of MEK on both S218 and S222 is required for its full activation, most of the MEK inhibitors were developed targeting one or both residues. For instance, the first FDA approved MEK inhibitor, trametinib (Figure 3A) for the treatment of metastatic melanoma was shown to selectively inhibit the phosphorylation of S218, but not S222 (Gilmartin et al., 2011). In general, MEK inhibitors are reported to act on unphosphorylated MEK or Raf/MEK complexes to prevent phosphorylation of MEK by Raf instead of inhibiting an active phosphorylated MEK.

A comparison of the co-crystal structure of the ATP analog, AMP-PNP (adenylyl imidodiphosphate) and to that of type-III allosteric inhibitors such as the approved drug cobimetinib shows that MEK1 adopts an inactive/locked/unphosphorylated conformation in which its αC-helix is shifted outward from its actual position (Figure 3B). This αC-helix out conformation is distinct from the αC-helix out inactive conformations of other kinases such as EGFR, AKT, or BRAF. The conformational change of the αC-helix forms a pocket adjacent to, but different from, the ATP-site in which the allosteric inhibitor binds (Figures 2, 3). Moreover, MEK1 also adopts similar autoinhibited αC-helix out conformations in the absence of an inhibitor. Thus, the co-crystal structures suggest that MEK1 inhibitors bind and stabilize a naturally occurring inactive conformation of the protein (Fischmann et al., 2009).

In addition to MEK1, type III allosteric inhibitors have been identified for other well-characterized kinases including AKT, EGFR, WNK (With-No-Lysine) and TRK (Tropomyosin receptor kinase) (Lu et al., 2020). The reported type III inhibitors are quite different for AKT and TRK in that they interact with domains beyond the kinase domain. AKT inhibitors interact at the interface of the kinase domain and the pleckstrin homology (PH) domain, whereas the TRK inhibitors interact at the interface of the kinase domain and the intracellular juxtamembrane region. While type III EGFR and WNK inhibitors bind exclusively at the kinase domain, like MEK1, their occupancy within the allosteric site significantly differs (Figure 3C).

The WNK family of kinases, consisting of WNK1-4, plays a significant role in regulating blood pressure and ion homeostasis (Wilson et al., 2001). In particular, the mutation in WNK family members causes pseudohypoaldosteronism type II, a rare Mendelian form of hypertension with hyperkalemia. In contrast to other kinases, the WNKs have an unusual placement of the catalytic lysine residue (K233 of WNK1) on the β2-strand in contrast to the β3-strand in all other kinases. It should be noted that the available rat *apo* crystal structure (6cn9) (Min et al., 2004) shows no sign of the presence of the type III allosteric pocket. The unique structural features resulting from the unusual position of the catalytic lysine led Yamada and coworkers to discover the first low nanomolar (<.01 µM) ATP-competitive pan-WNK kinase inhibitor, WNK463 (Yamada et al., 2016a). The published co-crystal structure with WNK463 (5drb (Yamada et al., 2016a)) revealed that the inhibitor occupies the back pocket of the catalytic site due to the unusual position of the catalytic lysine in the glycine-rich loop. Moreover, the WNK1-WNK463 binding complex structure closely resembles the *apo*-WNK1 structure, including the conformation of the A-loop.

Unfortunately, the advancement of WNK463 as a potential therapeutic was withdrawn due to its poor safety profile (Yamada et al., 2016a). In general, the development of WNK isoform selective inhibitors has been greatly hampered by the fact that their ATP sites are highly conserved. To circumvent this problem, the same group focused on less conserved regions of the WNK1 located outside the ATP pocket. By high throughput screening of a 1.2 million in-house compound collection, they identified several inhibitors with diverse scaffolds (Yamada et al., 2016b; Yamada et al., 2017). An X-ray crystal structure of WNK1 bound to the optimized WNK476 (5tf9, IC_50_ = .042 µM, Figure 3C) (Yamada et al., 2016b) demonstrated that it binds to the WNK1 type III allosteric site formed by the outward movement of the αC-helix and displacement of the A-loop (Yamada et al., 2016b). While there is some overlap between the binding modes of WNK476 and WNK463, there is no overlap in the binding mode of WNK476 and ATP, making it a true type III allosteric inhibitor. The discovery of WNK476 and subsequent crystal structure has led to a significant effort to find different and improved type III WNK inhibitors.

### Discovery of type III allosteric inhibitors by virtual screening

**FIGURE 4:**
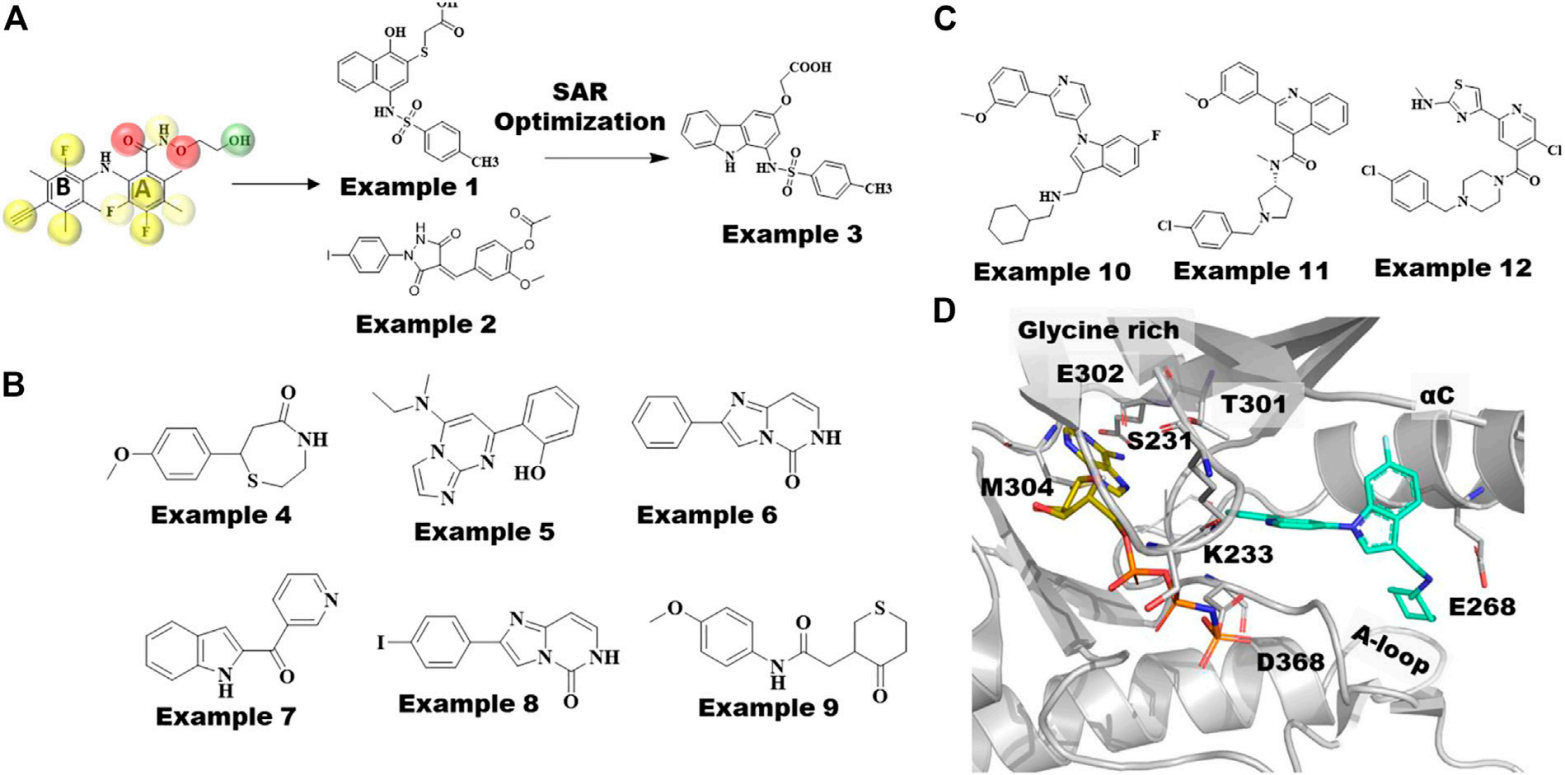
MEK and WNK Type III inhibitors discovered by computational techniques. **(A)** MEK1-Selumetinib structure(7MOT), categorized by hydrophobic pocket (L115, L118, V127, and M143) in cyan, catalytic lysine (K97) in green, and residues in the A-loop (C207DFGVS212, I215, M219) in red, involved in allosteric drug binding. The type III inhibitor, Selumetinib, is shown in yellow sticks, whereas novel non-biarylamine inhibitor RO5126766 is shown as megenta sticks. **(B)** Reported crystal structures of 11 MEK1 type III allosteric compounds possessing classic biarylamine scaffold used to build pharmacophore models in this study. Overlapped 2P55 ligand with its pharmacophore model, yellow spheres represent hydrophobic features, red spheres represent hydrogen bond acceptors, and green spheres represent hydrogen bond donors and their top 2 hit compounds example 1 (M100) and example 2 (M115) screened against Specs database optimized to get example 3 showed most potent inhibitory effect against Raf-MEK cascading. **(C)** Chemical structures of MEK fragment hits identified by Integrated Fragment and virtual screening. **(D)** Chemical structures of WNK1 allosteric inhibitors; examples 10, 11 and 12. Crystal structure of WNK1/AMP-PNP bound to allosteric inhibitor, example 10 is shown in cyan sticks and WNK1 represented as gray cartoon.

Most MEK inhibitors, including the 4 approved drugs, are structurally related biarylamines with similar binding modes (Figure 3). Comparison of MEK co-crystal structures show that although they bind to the same allosteric site, non-biarylamine inhibitors like RO5126766 (3wig) (Lito et al., 2014) adopt binding modes distinct from earlier biarylamines. Based on the structural knowledge gathered from MEK co-crystal structures, Xi et al. developed pharmacophore models consisting of 7–10 features from 11 reported crystal structures of MEK1-biarylamine complexes (Yamada et al., 2016b) (Figure 4A). The common pharmacophore features included six hydrophobic features, two hydrogen bond (HB) acceptors, and one HB donor along with 19 exclusion volumes. A pharmacophore-based screen against the Specs database with 200,158 compounds yielded 9,712 virtual hits. Subsequently, 13 chemically diverse drug-like compounds were tested for RAF-MEK1 inhibitory activity, of which 2 have shown moderate IC_50_ values of 27 µM (example 1, Figure 4A) and 26 µM (example 2, Figure 4A). Subsequent analogs produced numerous nM-level active compounds such as example 3 of Figure 4A, (Xi et al., 2019).

Fruscia and coworkers introduced a combination of computational and fragment-based approaches to identify novel allosteric MEK1 inhibitors (Di Fruscia et al., 2021). Interestingly, they first employed a library containing 15,000 fragments tailored to the MEK1 allosteric site using virtual screening methods. Subsequently, 1,000 fragments selected from docking calculations were employed in 1D NMR screening in the presence of an ATP analog to differentiate the allosteric binders. In total, 142 potential allosteric fragments were assessed by SPR, which were further narrowed down to six fragments. Of the six fragment hits shown in Figure 4B, examples 5 (K_d_ = 82 µM) and 7 (K_d_ = 65 µM), led to MEK1 cocrystal structures showing that these fragments bind to the same allosteric site as other type III allosteric inhibitors. Further substructure searches for close analogs of example 6 (K_d_ = 70 µM) led to example 8 (K_d_ = 28 µM). In addition, analogs of example 4 (Kd = 45 µM) yielded the most potent molecule example 9 (Kd = 0.03 µM), which also showed good bioavailability. High resolution co-crystal structures of both examples 8 and 9 revealed that the methoxy-phenyl group of example 9 and the iodo-phenyl group of example 8 occupy the same position as the 2,4-dihalogen group of cobimetinib (Figure 3B). Moreover, example 9 formed an additional polar contact with the catalytic residue D208 and water medicated contacts with S212 and V127, which were not observed in other allosteric MEK inhibitors. It would be interesting to apply this integrated fragment and computational screening in other kinases for identifying type III allosteric sites.

The binding mode of the type III allosteric inhibitor WNK476 of WNK1 (Figure 3C) resembles the binding mode of the MEK inhibitor PD3180883 (Ohren et al., 2004). In addition, both bind to similar kinase conformations, including DFG-in and αC-helix-out. Nevertheless, the residues that form the respective allosteric pockets are not conserved, and as such, obtaining selectivity across other kinases has not been an issue. Although the allosteric pocket is dissimilar from other kinase subfamilies, it shares high sequence similarity across the WNK subfamily. Yamada et al. utilized docking and small molecule alignments to optimize the potency and selectivity profile of the previously discovered allosteric compounds. Alignment of the crystal structure binding mode of WNK476 with the computational docking (Glide-SP v5.0 and MacroModel v9.6 (Schrödinger, LLC, New York)) pose of its novel core analogs allowed them to target a tight hydrophobic pocket around the aminothiazole/methoxyphenyl groups of WNK476 (Figure 3A). This pocket included potential novel interactions such as a hydrogen bond with V281 and a flexible hydrophobic pocket near the chlorophenyl/cyclohexylmethyl and a protonated amine linker for a potential hydrogen bond with E268. The crystal structure of WNK1 with AMP-PNP bound to example 10 (5wdy, IC_50_ = .039 µM, Figure 4D) (Yamada et al., 2017) revealed it to be an allosteric inhibitor with a novel scaffold binding mode as well as a hydrogen bond between E268 and its protonated amine linker. Furthermore, the exploration of co-crystal structures with other scaffolds, example 11 (IC_50_ = .75 µM, Figure 4D) (Yamada et al., 2017) led them to example 12 with an IC_50_ of .004 µM. More importantly, this compound showed ∼1,000-fold selectivity for WNK1 vs. WNK4 and 57-fold selectivity for WNK1 vs. WNK2 which is consistent with the residue differences around the allosteric site. Selectivity against WNK3 was not observed because the allosteric pocket lining residues are identical with those of WNK1. It is postulated that selectivity could be achieved focusing on amino acid differences in the highly flexible regions of the glycine rich loop and the αC-helix.

### Investigation of type III allosteric site by molecular dynamics simulations

Zhao et al. performed µ-second MD simulations to understand the behavior of MEK in the presence (4an2) and absence (3zls) of the approved type-III allosteric inhibitor drug cobimetinib (Figure 3A) (Zhao et al., 2017). They observed a reduction in the flexibility of the P-loop & A-loop, but a significant increase in flexibility of the αC-helix in the MEK1-cobimetinib complex compared to apo MEK. Interestingly, the study found that breaking the key salt-bridge between E114 of the aC-helix and the catalytic Lys97 led to the increased mobility of the αC-helix. This movement of the αC-helix has been recognized as a key component of the MEK1 allosteric binding site for type-III inhibitors. Most importantly, the A-loop forms a short helix in MEK1, which has also been implicated as a key element that allows the allosteric binding pocket to accommodate a type-III inhibitor. In comparison, for most kinases the A-loop is a flexible loop with little secondary structure. The authors analyzed all other kinases based on the allosteric pocket similarity and secondary structure prediction of the A-loop and concluded that only 15 other kinases, including MAST1/2/3, JAK3, MAP2K3/4/5/6, HPK1, HGK, TNIK, MINK, KHS1/2 and NRBP1, have the potential to be inhibited by type-III inhibitors. Notably, WNK1 also possesses a short helix in the A-loop that assists in forming the type III allosteric pocket near the αC-helix.

More recently, Fleischmann and coworkers developed a biosensor platform called KinCon to track different kinase conformational states in real time (Fleischmann et al., 2021). They applied this KinCon technology to record the MEK dynamics and subsequently confirmed it with MD simulations. Since the phosphorylation of MEK1 by RAF at the positions S218 and S222 in the A-loop promotes MEK1 activation, the KinCon experiment with the phosphomimic mutation on both phosphorylation residues induced an active conformation of the A-loop, disengagement of the N-terminal regulatory region and αC-helix, whereas MEK1 type-III inhibitor bound complexes were shown to promote a selective transition of the opened conformation to a more closed kinase state. To confirm the dynamic shifts, they also compared MD simulations on the crystal structures of Apo MEK1 (1sj9 and 3eqi) to those of ligand bound MEK1 (4u7z, 4lmn, and 3e8n). Further, simulations were performed on MEK1 where both phosphorylation residues were computationally mutated to either aspartic or glutamic acid. In total, thirteen simulations were performed for wild-type, mono or double mutant and with or without MEK1 type III an allosteric inhibitor. Intriguingly, the measured dynamics for MEK1 S218D/S222D double mutant exceeded the dynamics observed for the close analogous variant system: MEK1 S218E/S222E. Especially, compared to other variant systems, the MEK1 S218D/S222D double phosphomimic mutant exhibited a significant dynamic divergence on the distal A-helix and the αC-helix with broader distributions of the angles from its actual state. In contrast to double mutant, the presence of type III allosteric inhibitors in MEK1 specifically affects the αC-helix where double phosphomimic mutants induce a more pronounced conformational change compared to apo MEK1 or any MEK1 complex systems. Taken together, their KinCon experiment with the MD study showed that the binding of type III allosteric inhibitors to the allosteric site of MEK1 alters the αC-helix inducing a more closed state thereby inhibiting the activated/open state.

## Section—Protein tyrosine phosphatases

Protein tyrosine phosphatases balance the role of tyrosine kinases by catalyzing the removal of the phosphate group from previously phosphorylated tyrosine residues. Because phosphorylation plays a significant role in virtually every intracellular pathway, they are an important drug target class for many indications (Elhassan et al., 2021). While several tyrosine kinases, such as Jak1-3, EGFR and cABL, have been drugged, tyrosine phosphatases have yet to lead to an approved drug. The challenges with developing a drug for the orthosteric site of tyrosine phosphatases include the sequence conservation in their catalytic sites and their highly polar nature (Ghattas et al., 2016). As a result, allosteric inhibitors of tyrosine phosphatases are highly sought after.

**FIGURE 5:**
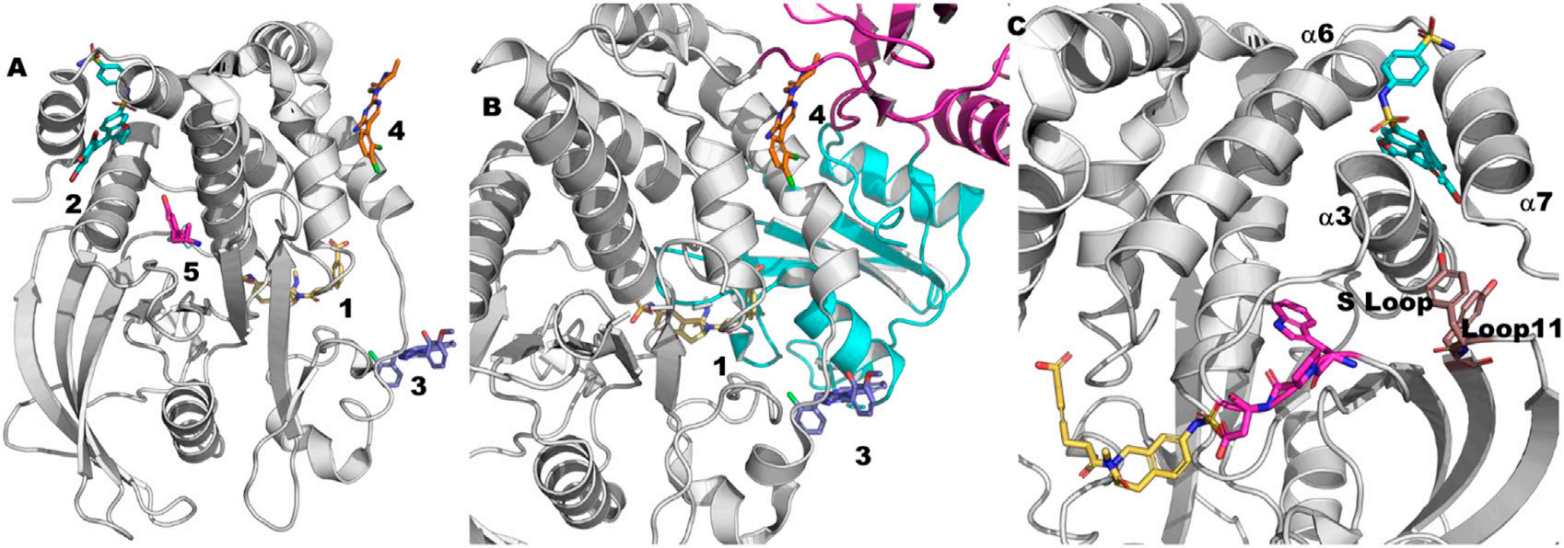
Tyrosine phosphatase ligands. **(A)** 1. An example of an orthosteric ligand (2f71 (Klopfenstein et al., 2006)). 2. The site of the allosteric PTP1B inhibitors (1t49 (Wiesmann et al., 2004)). This site is approximately 20–25 Å from the catalytic site. As is apparent from this picture this site is normally blocked by the helix referred to as a7. 3. An example of an SHP2 allosteric inhibitor that binds between the phosphatase domain and the N-SH2 domain of SHP2 (6bmr (Fodor et al., 2018)). This site is 10–15 Å from the catalytic site. 4. An example of an SHP2 allosteric inhibitor that binds between the phosphatase domain and the C-SH2 domain of SHP2 (5ehp (Garcia Fortanet et al., 2016)). This site is 15–20 Å. 5. A PTPN5 allosteric activator (6h8r (Tautermann et al., 2019)). This site is approximately 10 Å from the allosteric site (2) and 20 Å from the catalytic site. **(B)** The mechanism of action of the SHP2 allosteric inhibitors. The N-SH2 domain is shown in cyan. The C-SH2 domain is shown in magenta. The phosphatase domain is shown in green. The compounds are numbered as in part **(A) (C)** The gray ribbon is PTP1B as bound to an orthosteric inhibitor (yellow) from the structure 2f71 (Klopfenstein et al., 2006). The cyan ligand is the allosteric inhibitor from the co-crystal structure 1t49 (Wiesmann et al., 2004). a7 is not visible in this cocrystal structure presumably due to the presence of the allosteric inhibitor. Y152-Y153 are shown as sticks in brown. The WPD motif (179-181) is show in magenta.

Allosteric inhibitors have been extensively pursued for two of the family members: SHP2 and PTP1B. Despite having structurally similar phosphatase domains, the modes of allosteric inhibition of these two targets are very different (Figure 5). The known PTP1B allosteric inhibitors bind at a site (site 2 of Figure 5A) that is approximately 20 Å from the catalytic site. By binding at this site, these compounds ultimately induce a conformational change in the loops comprising the catalytic site sufficient to disrupt its phosphatase activity. SHP2 differs from PTP1B in that it has a pair of N-term SH2 domains. In its autoinhibited form, the two SH2 domains bind to its phosphatase domain and block its catalytic site (Garcia Fortanet et al., 2016; Fodor et al., 2018). When the two SH2 domains bind to the appropriate phospho-tyrosine peptides, they undergo a conformational change that disrupts their binding to its phosphatase domain, thereby relieving the autoinhibition and allowing SHP2 to become a functioning phosphatase. The available SHP2 allosteric inhibitors bind at the interface of the phosphatase and one of the SH2 domains, thereby locking SHP2 in its autoinhibited form (Figure 5B). Indeed, allosteric inhibitors of SHP2 have progressed into clinical trials (Song et al., 2022), whereas the PTP1B allosteric inhibitors have stalled in large part due to insufficient potency. It is the challenges associated with the latter that we discuss here.

### The role of molecular dynamics in understanding PTP1B allosteric inhibitors

Allosteric PTP1B inhibitors were first reported in 2004 (Wiesmann et al., 2004). The mechanism of allosteric inhibition was studied *via* molecular dynamics as early as 2006 (Kamerlin et al., 2006) and many times since (Kamerlin et al., 2007; Bharatham et al., 2008; Kumar et al., 2010; Olmez and Alakent, 2011; Bakan et al., 2012; Cui et al., 2013; Eren and Alakent, 2013; Shinde and Sobhia, 2013; Choy et al., 2017; Hjortness et al., 2018; Torgeson et al., 2020; Crean et al., 2021). One of the earliest conclusions from these studies is that the presence of an allosteric inhibitor reduces the flexibility of the loops in the catalytic site, in particular the critical WPD loop (Kamerlin et al., 2007) (residues 179–183) that must move from an open/inactive state to a closed/active state during catalysis. Subsequently, full allosteric pathways have been proposed *via* molecular dynamics which begin with allosteric inhibitors at this site disrupting the binding of a7 (residues 286–298) (Olmez and Alakent, 2011; Li et al., 2014) to neighboring helices a3 (residues 188–201) and a6 (residues 264–279) (Figure 5C). For example, Kamerlin et al. (2006) and Kamerlin et al. (2007) used short (2 ns) simulations in which they steered the WPD loop from the open to closed form and *vice versa*. They found that the S-loop (residues 198–209) transmits the signal to a3 and finally to a7. They subsequently found that the presence of a bound allosteric inhibitor decreased the flexibility of both the S-loop and the WPD loop.

While subsequent experimental work is generally consistent with this model, more details have been revealed in the last decade that warrant more detailed molecular dynamics studies. For example, in separate NMR studies Cui et al. (2017) and Choy et al. (2017) reveal the importance of loop 11, in particular Y152 and Y153 of loop 11 (Figure 5C), in connecting the mobility of a7 to the catalytic activity of PTP1B.

It is worth noting that the only other tyrosine phosphatase with the equivalent of a7 is the T-cell protein-tyrosine phosphatase (TCPTP), which may explain the selectivity of this class of inhibitors across the phosphatase family. Recently, it has been shown that TCPTP also depends on the stable binding of a7 to achieve its maximal catalytic efficiency (Singh et al., 2021). A second area where dynamics could shed further light on the allosteric mechanism and potentially lead to new approaches to allosteric inhibitors is to understand why PTP1B and TCPTP require a7 for optimal efficiency whereas the rest of the family lacks this helix altogether.

### PTP1B druggability assessment

The biggest challenge with optimizing current PTP1B allosteric inhibitors has been achieving the necessary potency. Indeed, despite extensive effort, breaking the 1 µM barrier with these compounds has been difficult. This raises an important question for PTP1B which is likely be common to many allosteric campaigns: which allosteric sites are amenable to drug-like potency along with the necessary allosteric effects on the orthosteric site? Thus, PTP1B provides a good challenge for druggability assessment at both the orthosteric and allosteric sites.

Bakan and coworkers use PTP1B along with several other proteins to describe a simulation based approach to druggability assessment (Bakan et al., 2012). Briefly, for their approach, they selected several small organic probes: isopropanol, acetamide, acetic acid and isopropylamine. The fragments were used simultaneously in an explicit solvent MD simulation. Occupancy grids were then calculated for each fragment from the simulation trajectories. The occupancy grids are in turn used to generate binding free energy grids using Boltzmann’s equation. These free energy grids were then used to estimate the maximal affinity achievable at a given site. This approach to druggability might be particularly valuable for allosteric sites because it is better able to incorporate the necessary protein flexibility than traditional methods based on static crystal structures.

For PTP1B, their calculations yielded a maximal affinity of .0003–.0009 μM at the orthosteric site. Because the catalytic site has evolved in large part to recognize a phosphate, the acetate probe is the dominant probe, as expected. In comparison, the calculated maximal affinity at the allosteric site is in the range of 9–18 µM. Because the allosteric site is largely hydrophobic, isopropanol was the dominant fragment. Further, the allosteric site of PTP1B had the lowest calculated maximal affinity of all the proteins/sites studied: for the other proteins considered, MDM2, LFA, EG5, and p38, the next weakest maximal affinity, .047 µM, is for a 3rd site on EG5. It is noteworthy that many of the sites in the other proteins used in this study were also allosteric. Thus, this approach predicts that gaining sufficient affinity at the PTP1B allosteric site is difficult with the caveat that the starting structure for the PTP1B simulation did not have a7 and leaves open the possibility of a compound cooperatively interacting with a7 rather than displacing it showing greater potency.

**FIGURE 6:**
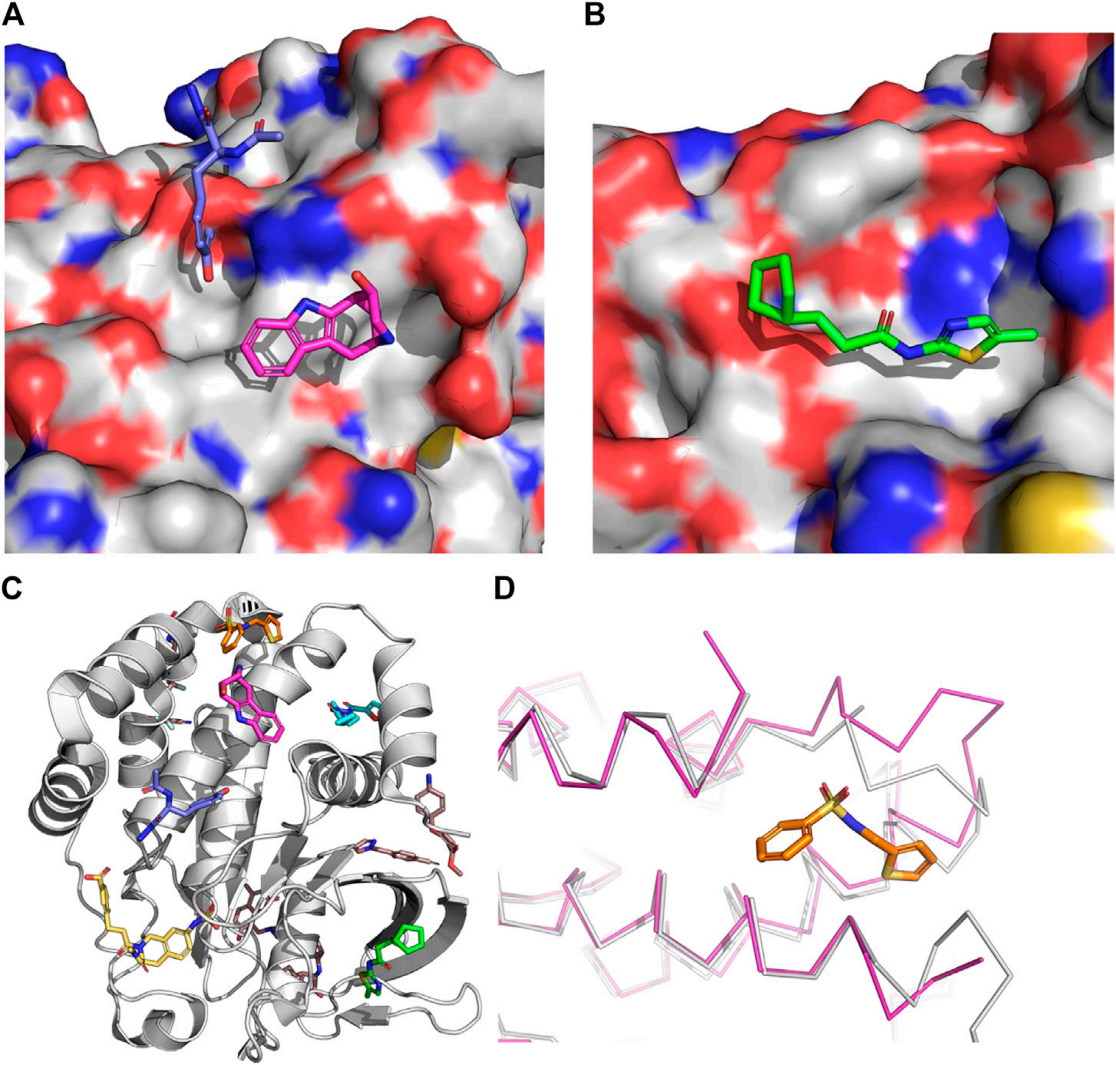
The surface is that of PTP1B from the structure 2f71. **(A)** Insulin receptor kinase (IRK) site. The co-crystallized fragments are from the structure 5qg8 (Keedy et al., 2018) (magenta) and 5qdx (blue). **(B)** Site 4. The co-crystallized fragment is from the structure 5qee (Keedy et al., 2018). **(C)** Examples from each site found in the fragment-based crystallography effort by Keedy and co-workers (Keedy et al., 2018). **(D)**. The largest cluster of fragments found outside the orthosteric or initial allosteric site. The magenta ribbon is PTP1B as bound to this fragment (from 5qdl (Keedy et al., 2018)) whereas the gray ribbon is PTP1B as bound to an orthosteric inhibitor. The loop with the largest deviation is residues 236–244 with an RMSD of 2.9Å and a maximum deviation over 6 Å. This demonstrates the need for significant induce fit to identify this as a potential site to target.

Surprisingly, their approach highlighted two additional sites on PTP1B that are predicted to have greater maximal affinities than the known allosteric site: the IRK (insulin receptor kinase) interaction site (maximal affinity of .043 µM, Figure 6A) and a fourth site (maximal affinity = .2 µM, Figure 6B) to which there were no compounds known to bind. Both of these sites were subsequently identified from a fragment-based crystallography effort (Keedy et al., 2018). In this work Keedy and colleagues combined multi-temperature crystallography with high throughput fragment soaking. In all they resolved 110 fragments in complex with PTP1B which fall into 11 binding sites outside the catalytic site (Figure 6C). The IRK site was occupied by only 1 of the fragments, while the fourth site was occupied by 2 separate clusters of fragments, one containing 8 fragments and the other 3 fragments. While it remains to be seen whether any of these new sites can lead to potent PTP1B modulators, it highlights the challenge of handling induce fit properly. The site other than the orthosteric site with the largest number of bound fragments found crystallographically was missed by the computational study. This appears to be due to the significant amount of induced fit observed when the fragments bind at this site (Figure 6D).

Recently a fragment allosteric activator was discovered for Striatal-Enriched Protein Tyrosine Phosphatase, also known as PTPN5 (Tautermann et al., 2019) (see site 5 of Figure 5A). As with the examples found *via* the fragment-based work for PTP1B described in the previous paragraph, it remains to be seen if these molecules can be improved to have drug-like potencies. The most potent example thus far has a binding affinity *via* ITC of 38 µM. It is noteworthy that none of the 110 fragments co-crystallized with PTP1B described in the previous paragraph occupied the site equivalent to that of PTPN5 occupied by this activator fragment. It is likely that even though PTP1B and PTPN5 are highly related, the mechanism of this allosteric activator is unique to PTPN5.

## Section—Nuclear hormone receptors

The nuclear hormone receptors (NHRs) are a family of transcription factors consisting of 48 members. They are central players in many physiological processes and have been the targets for numerous drug discovery efforts and ultimately approved drugs (Rask-Andersen et al., 2011; Tice and Zheng, 2016). Most of this effort has focused on molecules that mimic their natural ligands, i.e., target their orthosteric site. This site has several advantages. First, the volume of the site is consistent with a drug-sized molecule and has a good hydrophobic/hydrophilic balance to yield potent drug-like small molecules often covering many distinct chemotypes. Second, the site is well known to yield both agonists and antagonists. Often small changes in structure will convert one member of a chemical series from an agonist to an antagonist and *vice versa*. It is noteworthy that even though it is referred to as the orthosteric site, the functional consequences of orthosteric ligands are determined by how they direct the conformation of helix 12, which in turn is critical for interacting with its various coactivators.

**FIGURE 7:**
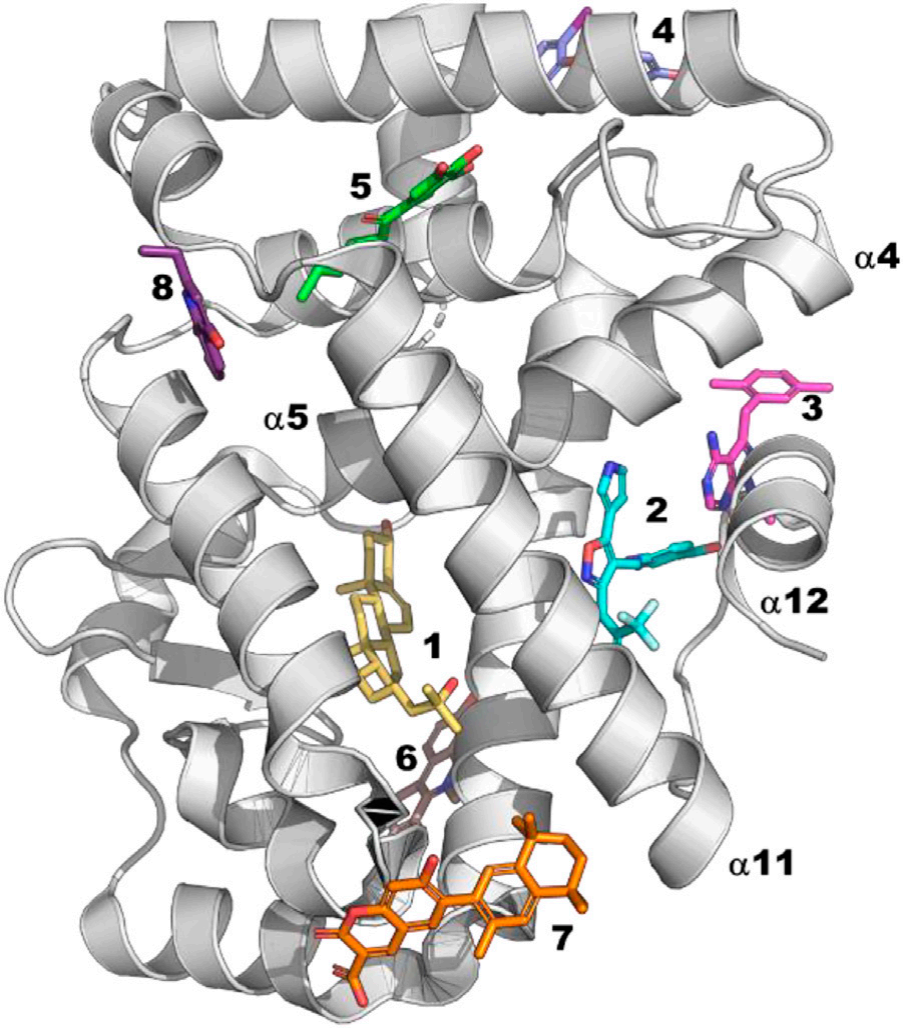
Site 1: Orthosteric site for which there are many examples. Site 2: RORg allosteric inverse agonists (6t4x (de Vries et al., 2021)), RXRg (5tbp (Chen et al., 2017)). This ligand causes an N-term isoform common in cancer cells to tetramerize but has no effect on full length. Site 3: Androgen receptor allosteric antagonist (2pip (Estébanez-Perpiñá et al., 2007)). Site 4: Androgen receptor allosteric antagonists (2pit (Estébanez-Perpiñá et al., 2007)). Site 5: TR3 (4ref (Wang et al., 2015)). Site 6: PPARa (7c6q). Site 7: RXRa (6jno (Yamada et al., 2019)). Site 8: RORg (5g46 (Xue et al., 2016))—fragment found by crystallographic screening.

Because NHRs directly interact with numerous partners that regulate their function, they offer the potential to find modulators outside their traditional orthosteric site (Moore et al., 2010). Indeed, many NHR crystal structures have been solved with ligands at sites other than the orthosteric site (Figure 7). Perhaps the most successful effort to find allosteric modulators within the NHR family is RORg. Allosteric inverse agonists were first described for RORg by Scheepstra et al. (2015). The compounds are remarkable in that they force helix 12 into a position close to that of a traditional orthosteric antagonist while binding at a distinct site (Site 2 in Figure 7). Despite the intensive drug discovery efforts focused on the NHR family, this site has only been observed in one other family member, RXRa (5tbp (Chen et al., 2017)), with very different structural consequences (tetramer stabilization). This raises the question: what is different about RORg that allows for this binding mode?

**FIGURE 8:**
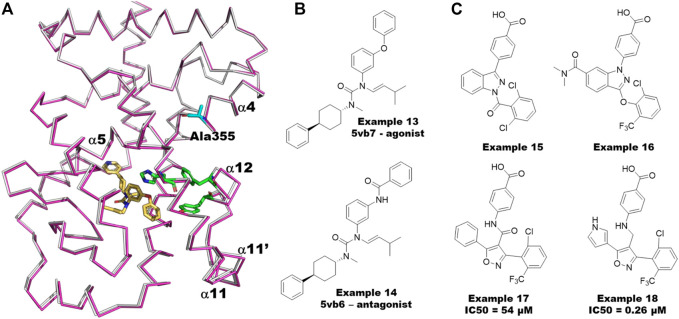
Allosteric Modeling Examples for RORg. **(A)** The matched agonist/antagonist pair, examples 13 and 14, from the co-crystal structures 5vb6 and 5vb7 (Li et al., 2017) respectively. **(B)** A structural overlay of the two co-crystal structures. RORg from 5vb6 (antagonist bound) is shown in grey and that from 5vb7 (agonist bound) in magenta. The HYF triad (H479-Y502-F506) is shown as green sticks. A355 is shown as cyan sticks. The agonist from 5vb7 is shown in yellow. The main helices (4, 5, 11, 11′ and 12) are numbered according to their use in the text. **(C)** Examples 15 and 16 are the RORg inverse agonists used to build the pharmacophore model. Example 17 is the initial hit from the virtual screen/design. Example 18 is the most potent molecule from the follow up synthesis.

Consistent with the fact that RORg shows considerable basal activity, an observation from the many co-crystal structures of RORg is that helix 12 is stabilized in the active conformation even without a bound agonist. Through an analysis of RORg crystal structures, Li and coworkers (Li et al., 2017) identified features of RORg that are different from other NHRs and likely contribute to its unusual behavior. First, the active conformation of helix 12 is stabilized by a unique triad of interacting residues involving H479, Y502 and F506 (HYF triad). H479 is located on helix 11 while Y502 and F506 are located on helix 12 (Figure 8A). To support the significance of the observed interactions they calculated the pairwise interaction energy between the residues of the triad to be −12.9 kcal/mol. Further they point out that the RORg isoforms are the only members of the NHR family that have this triad of residues: the closest being PPARg which has a corresponding HLY motif, residues 255–257, resulting in only -3.0 kcal/mol interaction energy. Second, a small helix, termed helix 11′, is observed between helix 11 and helix 12 of RORg but virtually non-existent in other NHRs. As helix 11′ packs against helix 12 in the active form of RORg, it likely contributes to stabilizing helix 12 in the active conformation.

### Application of molecular dynamics in understanding RORg allostery

Using molecular dynamics, Yuan et al. (2019) shed further light on the unique behavior of RORg. They performed molecular dynamics simulations of 4 different RORg systems: *apo* (5vb3 (Li et al., 2017)), agonist bound (5vb7 (Li et al., 2017)), inverse agonist bound (5vb5 (Li et al., 2017)) and allosteric inverse agonist bound (5c4o (Scheepstra et al., 2015)). Their calculations support both the importance of the HYF triad and the hinge between helix 11 and helix 12 as reported by Li and coworkers.

In a second molecular dynamics study, Saen-Oon et al. (2019) compared the dynamics of several different states of the system including the agonist bound, orthosteric inverse agonist bound and orthosteric antagonist bound systems. First, they find that the bound agonist stabilizes the HYF triad: the H479-Y502 hydrogen bond is broken 12.5% of the time with the *apo* simulation compared to only <.1% of the time for the agonist bound structure. This is consistent with the observation that RORg has basal activity but increased activity in the presence of agonist. They further elucidate the significance of the HYF triad by comparing simulations with examples 13 and 14 which are matched agonist/antagonist pairs (Figure 8B). Despite the functional difference between the two molecules, their cocrystal structures are nearly identical (Li et al., 2017) For example the Cα RMSD is less than .2 Å (Figure 8A). Despite the similarity of the two compounds and similarity of the co-crystal structures, the simulations show that the agonist further stabilizes the HYF triad interaction while the antagonist significantly disrupts the interaction both as measured by the length of the Y502-H479 hydrogen bond and the total interaction energy of the HYF triad. In the case of the agonist, the hydrogen bond distance stays very close to 3.0 Å whereas the distance averages 8.0 Å over the course of the simulation with the antagonist bound. Further, the pairwise interaction energy of the HYF triad during the simulation with RORg bound to the agonist averages −8 kcal/mol compared to near 0 for the antagonist bound simulation.

In a recent study, de Vries and co-workers (de Vries et al., 2021) use a variety of experimental techniques along with MD to quantify and understand the cooperativity of simultaneous ligand binding between the orthosteric and allosteric sites of RORg. To demonstrate cooperative binding, they measured the thermal shift with ligands in combination compared to the ligands alone. The orthosteric agonist 20a-hydroxy-cholesterol (20-OH) causes an increase in RORg thermal stability of 3.6°C. Using 3 allosteric inverse agonists that lead to increases in thermal stability ranging from 1°C to 7°C they find an additional increase in thermal stability of approximately 6°C–8°C when combined with 20-OH suggesting cooperative binding. By solving a variety of co-crystal structures, they further observe a shift in helix 4 in the presence of orthosteric ligands. They used molecular dynamics simulations with a variety of ligands and combinations of ligands to identify the unusual characteristic of RORg that may explain its ability to cooperatively bind orthosteric and allosteric ligands: namely that A355 can switch between helix 4 and helix 5 and that in the presence of ligands at both sites has a strong preference to be part of helix5. Therefore, they propose that the extent to which an orthosteric ligand biases A355 (Figure 8A) to helix 5 dictates the extent of its binding cooperativity with the orthosteric site.

### An application of virtual screening and structure-based design to the discovery of new RORg allosteric inverse agonists

Meijer et al. (2020) describe a virtual screening approach that led to the discovery of a new series of sub-micromolar RORg allosteric inverse agonists. As a first step they used a 6-point Phase (Dixon et al., 2006) pharmacophore model built based on inverse agonists (examples 15 and 16 in Figure 8C) to screen approximately 290,000 molecules from the Asinex virtual collection. Of the 30 top hits, 13 were of the same scaffold but matched only 4 of the 6 desired pharmacophore features. Accordingly, 2 compounds were designed and synthesized with the same scaffold but matching 5 of the 6 features. One of the two compounds (example 17, Figure 8C) demonstrated an IC_50_ of around 50 µM in a TR-FRET coactivator recruitment assay. Subsequent docking of a small virtual library and synthesis of a small number of analogs led to a series of RORg allosteric inverse agonists as potent as .26 µM (example 18, Figure 8C).

## Section—Peptidases—Cathepsins and other related peptidases

Cathepsins are members of the papain superfamily of cysteine proteases. They are generally localized within the lysosomes where they have key roles in functions such as protein degradation, autophagy, cell death and more (Novinec et al., 2014a; Olson and Joyce, 2015; Berdowska et al., 2021; Gaire et al., 2021; Yoo et al., 2022). In most situations, the proteolytic activity of cathepsins is restricted to the acidic environment within the lysosomes (Verma et al., 2016). In addition, they are reported to be frequently overexpressed in tumors that led to a plethora of cancer research studies investigating the role of cathepsins and cancer (Oskarsson, 2013). This connection to cancer added support to their extra-lysosomal localization and activity due to the acidic nature of the extracellular environment in tumors (Kato et al., 2013). Further, non-proteolytic collagenase activity has been proposed for the extra-lysosomal cathepsins in the extracellular matrix (Novinec and Lenarčič, 2013; Novinec et al., 2014b). Very recently, cathepsin L was reported to be involved in the activation of SARS-CoV-2 spike protein within the gastrointestinal tract (Berdowska et al., 2021).

The human cathepsin family comprises 11 members of which the majority are endopeptidases (B, F, H, K, L, O, S, V, and W): cathepsin B and X are also carboxypeptidases, H is also an aminopeptidase, and C is a dipeptidyl peptidase (Olson and Joyce, 2015). All cathepsins are synthesized as inactive proenzymes (zymogens), where a peptide (referred to as the propeptide) covers the catalytic site of the enzyme (Novinec et al., 2014a). Removal of the propeptide is required for the full proteolytic activity of the enzyme, which adds considerable complexity to the mechanistic understanding of the cathepsin activation. Multiple mechanisms have been proposed for the removal of the propeptide. The simplest mechanism explains that either the propeptide is cleaved by other proteases or auto-cleaved in the low pH environment of lysosome (Rozman et al., 1999; Pungerčar et al., 2009). Other structural studies claimed that the propeptide binds the catalytic site opposite to the direction that a substrate would bind, suggesting that an autoactivation mechanism may not be possible. Another study on cathepsin B suggested that autoactivation is accelerated in the presence of active cathepsin B molecules indicating a bimolecular process (Rozman et al., 1999) but the initiation of the process was not described. The standing and most accepted explanation is based on the mutational studies of the linker that connects the propeptide to the rest of cathepsin B^93^. It describes the mechanism as a two-step process: i) low pH-induced conformational changes in the propeptide partially exposes the catalytic site of a procathepsin B, which is a unimolecular event and ii) two procathepsin B molecules with partially exposed catalytic sites come in proximity and cleave the other propeptide.

**FIGURE 9:**
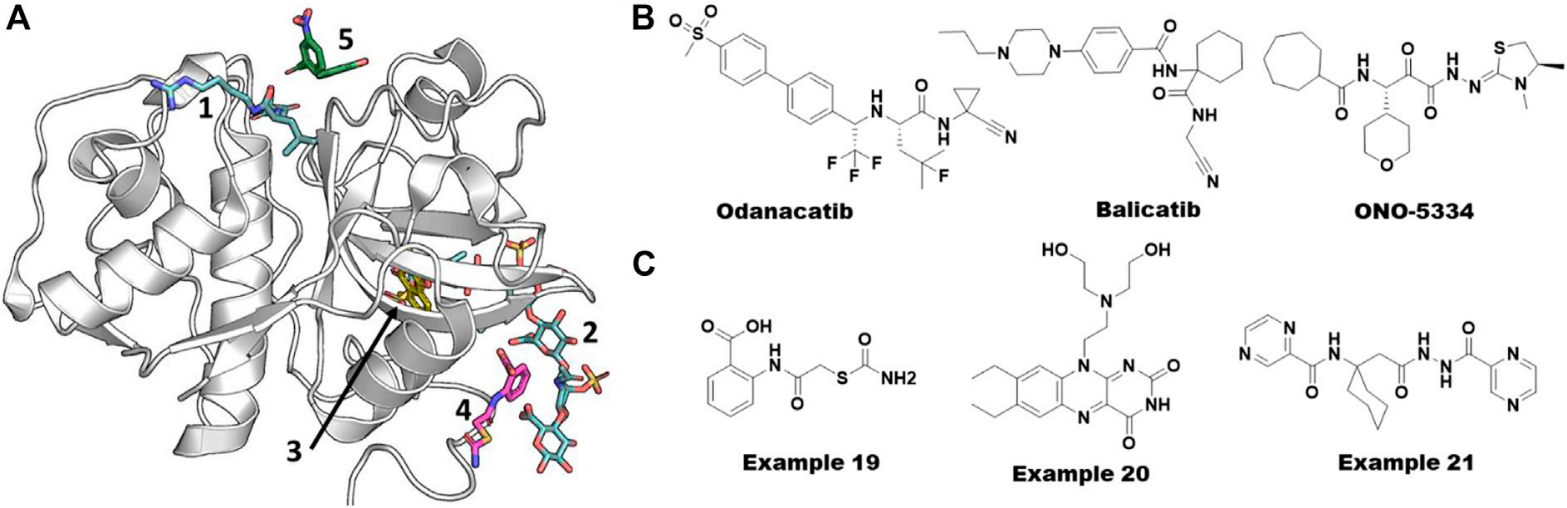
Allosteric ligands of cathepsin K. **(A)** Different pockets including orthosteric catalytic pocket, allosteric, and ectosteric pockets. Various x-ray structures were overlaid on to PDB ID. 5j94 to show different pockets bound with ligands. Catalytic and GAG site ligands are shown from PDB ID. 3h7d. Another allosteric site binder bound in three sites (blue from PDB ID. 6ash). Ectosteric site binders (yellow gold color) are bound at multiple shown from PDB ID. 6pxf. The catalytic site binder in facipain is bound slightly differently compared to cathepsin K. **(B)** Small molecules failed in clinical stages for various reasons targeting Cathepsin K. **(C)** Allosteric small molecule inhibitors of Cathepsin K obtained from virtual screening. Their reported IC50 values were 80 µM (example 19) and 300 nM (example 20) from substrate hydrolysis assay and osteoclast resorption assay, respectively. Azapeptide hit of Falcipain (example 21) abolishing the growth the of malarial parasite *via* blocking the maturation process.

In addition to the low pH conditions, binding of glycosaminoglycans (GAGs) have also been shown to promote the activation of cathepsins even at pH 6.5^92^. In fact, chondroitin sulfate (CS) and other GAGs allosterically bind cathepsin K with different affinities and strongly couple to its collagenase activity (Li et al., 2008; Novinec et al., 2010; Novinec et al., 2014a). GAG concentrations in normal bone were reported to be sufficient for cathepsin K to achieve its full collagenase activity. Structural information of allosteric pockets in cathepsins come from the crystal structures of cathepsin K (Figure 9A). In addition to the allosteric sites, collagenase-specific multiple ectosteric sites have also been identified and targeted. These ectosteric sites are different from the allosteric sites in that they do not affect the orthosteric site responsible for the catalytic activity of the enzyme (Law et al., 2017).

Cathepsin K is considered a promising target for the treatment of osteoporosis due to its key role in bone turnover and remodeling. Due to this function, pharmaceutical companies have worked on the development of cathepsin K inhibitors, but none of these inhibitors have been approved by the FDA to date (Dai et al., 2020). Among the most promising clinical candidates, odanacatib from Merck & Co. failed in Phase III clinical trials due to the cardio-cerebrovascular side effects. Balicatib, developed by Novartis, failed in Phase II trials because of unexpected skin lesions. After successful Phase I and II clinical trials, development of ONO-5334 from Ono Pharmaceuticals Co. was discontinued in the osteoporosis area due to the competitive situation in the osteoporosis area and changes in the environment. See Figure 9B for the structures of these molecules. Falcipain, a prokaryotic homolog of cathepsins, has also been reported as a key target for developing anti-malarial therapies. It has a similar structural fold and therefore likely utilizes the same allosteric sites reported for cathepsins (Marques et al., 2013).

### Identification and conformational flexibility of allosteric sites in cathepsins

The complexity in understanding the mechanistic details of cathepsins is manifold. First, as described in the previous section, their propeptides must be cleaved for activation. Second, their collagenase activity is modulated *via* allosteric sites by GAGs. Finally, their ectosteric sites specifically control its collagenase activity without affecting the catalytic site. Novinec and coworkers contributed significantly to the identification and understanding of allosteric sites in cathepsins employing both experimental and computational approaches. They discovered the first small molecule collagenase inhibitor, example 19 (Figure 9C), from a high-throughput docking study by sampling compound libraries at computationally identified sites from an evolution-based statistical coupling analysis (SCA) (Novinec et al., 2014b; Lu et al., 2014).

In their pioneering work in 2009, Halabi et al. (2009) introduced a concept of “protein sectors” as an organization of protein structural elements beyond the hierarchy of primary, secondary, and tertiary structural features. This concept was first applied to the S1A family of serine proteases and proposed that the “protein sectors” could represent the history of evolution of conserved properties and a “wiring” that can rapidly gain control over the function of a protein. Results from calculating these sectors using SCA on cathepsin K revealed a spatial distribution of sector residues displaying a continuous network around the active site and expanding throughout the protein. They used the *AutoLigand* tool (Harris et al., 2007) to predict allosteric pockets and filtered based on protein sectors. One of the predicted pockets represented the previously known CS binding site and hence validated the results. They predicted eight pockets in total of which two sites were deeper cavities, four were shallower, one was flatter with protruding loops, and the other was the CS binding pocket.

Novinec et al., combining experimental and computational approaches, showed that the conformational flexibility of cathepsin K is significantly dependent on plasma pH. The *in vitro* kinetic measurements showed that the enzyme exists in multiple distinct conformational states with different kinetic properties. The GAGs and other small molecule binders leverage this conformational flexibility to bind and regulate the enzyme function (Novinec et al., 2010). Despite the wealth of structural information obtained from hundreds of crystal structures of all cathepsins, conformational dynamics of the enzyme has been missing. In 2017, Novinec addressed this issue using an ensemble of MD simulations of *apo* and bound forms of cathepsin K with different small molecules such as example 19 (Figure 9C) and others (Novinec, 2017). They concluded that the small molecule binders stabilize a particular conformational state of the enzyme and thereby inhibit substrate binding. Recently, Rocha et al. (2020) used MD simulations and correlation networks to evaluate the potential of well-established allosteric pockets in cathepsin K to communicate to the catalytic site . Though their work remained purely computational, their results confirmed the previous experimentally known GAG binding site as one of the possible allosteric sites.

### Successful virtual screening campaigns on cathepsin K and falcipain

After the recent failure of odanacatib, to our knowledge there are no small molecules in active development for modulating the functions of cathepsin K. In this section, we discuss the virtual screening efforts to identify small molecule modulators for cathepsin K and falcipain, the malarial homolog of cathepsins, as it is out of scope of this review to cover all 11 members of the family. One of the successful virtual screening efforts was performed by Novinec et al. using high-throughput docking of compound libraries to the allosteric sites, which resulted in example 19 (Figure 9C) as discussed in the previous section (Novinec et al., 2014b).

Building on their site and structural analysis described above, Novinec and colleagues screened diverse compound libraries against several identified allosteric sites using a two-step docking method, where the complete library was docked with a high-throughput setup using *UCSF DOCK* (Allen et al., 2015) followed by docking of only the top 10% of the hits using the *AutoDock* program (Morris et al., 2009). The hits found from this docking protocol were screened experimentally using proteolytic and collagenolytic assays which resulted in 15 compounds (including example 19, Figure 9C) showing an effect on cathepsin K activity. Co-crystallization of example 19 successfully provided the first structural information of an allosteric site of cathepsin K (PDB 5j94) (Novinec et al., 2014b). In a follow-up study using site-directed mutagenesis, the same group added evidence for the direct involvement of this allosteric site in modulating the binding of GAGs and other synthetic scaffolds (Novinec et al., 2016).

In the second example of a successful virtual screen for allosteric inhibitors, Rocha et al. have identified potential selective ectosteric site binders from a computational exploration of ∼14,000 druglike compounds available at the Chemical Repository of the National Cancer Institute-Development Therapeutics Program (NCI-DTP) (Law et al., 2017). They used a composite docking score that combined results from three docking programs (Surflex, Glide, and GOLD) to evaluate the binding potential of a compound. They claimed that the composite docking method surpassed the individual methods by 5-fold in identifying potent inhibitors. Subsequent experimental testing of the hit compounds showed inhibitory effects on the bone resorption with the lowest IC_50_ value of .3 µM (example 20, Figure 9C) without cell toxicity (Law et al., 2017).

All other virtual screening efforts have focused on catalytic site binders including covalent binders for cathepsin K. Wang et al. and Ravikumar et al. have performed a virtual screen combining a ligand-based pharmacophore model and molecular docking for the catalytic site (Ravikumar et al., 2008; Wang et al., 2016). Schröder et al. (2013) have used docking-based virtual screening and identified a carbonitrile compound with a Ki value of .021 µM, which was confirmed to have a covalent reversible mechanism of inhibition. Stumpfe et al. (2010) have developed the *DynaMAD* algorithm combining mini-fingerprint searching and compound mapping methods to identify two selective inhibitors for cathepsin K.

Alberca et al. have tried repurposing the clinically failed odanacatib (Figure 9B) and an antibiotic methacycline for the inhibition of hemoglobinase activity of falcipain-2 with reported K_i_ values of .098 µM and 84 μM, respectively (Alberca et al., 2019). They also reported that methacycline is a non-competitive inhibitor. Gonzalez et al. have postulated that a previously known non-competitive chalcone inhibitor of falcipain-2 might be binding to a transient pocket mostly occluded in the crystal structures using molecular docking and MD simulations combined with free energy calculations (Hernández González et al., 2019). In another study, Pant et al. targeted an allosteric site between the propeptide and mature falcipains during autoprocessing. The combination of ensemble molecular docking, MM-PBSA, and accelerated ligand sampling MD simulations was employed to evaluate their library of azapeptide compounds. Two compounds were experimentally shown to inhibit the growth of the parasite by inhibiting the falcipains allosterically (EC_50_ = .8 µM) with no cytotoxicity (example 21, Figure 9C). Altogether, these potential allosteric pockets added new avenues to design potent inhibitors for this difficult to target protein family of peptidases.

## Section—Protein arginine methyl transferases

Protein arginine methyltransferases (PRMTs) are a family of enzymes that transfer the methyl group from S-adenosylmethionine (SAM) to one or two of the guanidine nitrogens of an arginine residue of a substrate protein. PRMTs participate in several cellular processes, including phase separation, DNA damage repair, transcriptional regulation, and RNA metabolism. Hence, the PRMTs have a profound effect on human diseases such as cancer and cardiovascular diseases (Bouras et al., 2013). In particular, the overexpression of PRMTs have been described in numerous cancers (Poulard et al., 2016). To date, nine PRMTs (PRMT1-9) have been identified in humans, which are further classified into three types of arginine methylations based on the catalytic mechanism. Type I PRMTs, including PRMT1, 2, 3, 4, 6, and 8, catalyze the formation of monomethylarginine (MMA) and asymmetric dimethylarginine (ADMA). Type II PRMTs, including PRMT5 and PRMT9, catalyze the formation of MMA and symmetric dimethylarginine (SDMA). PRMT7 is the only type III PRMT which solely generates MMA (Bedford and Clarke, 2009; Fuhrmann et al., 2015). Because the functions of PRMTs are governed by their substrates and regulators, the family has the potential for both orthosteric and allosteric modulators.

### Classified allosteric sites of PRMTs

**FIGURE 10:**
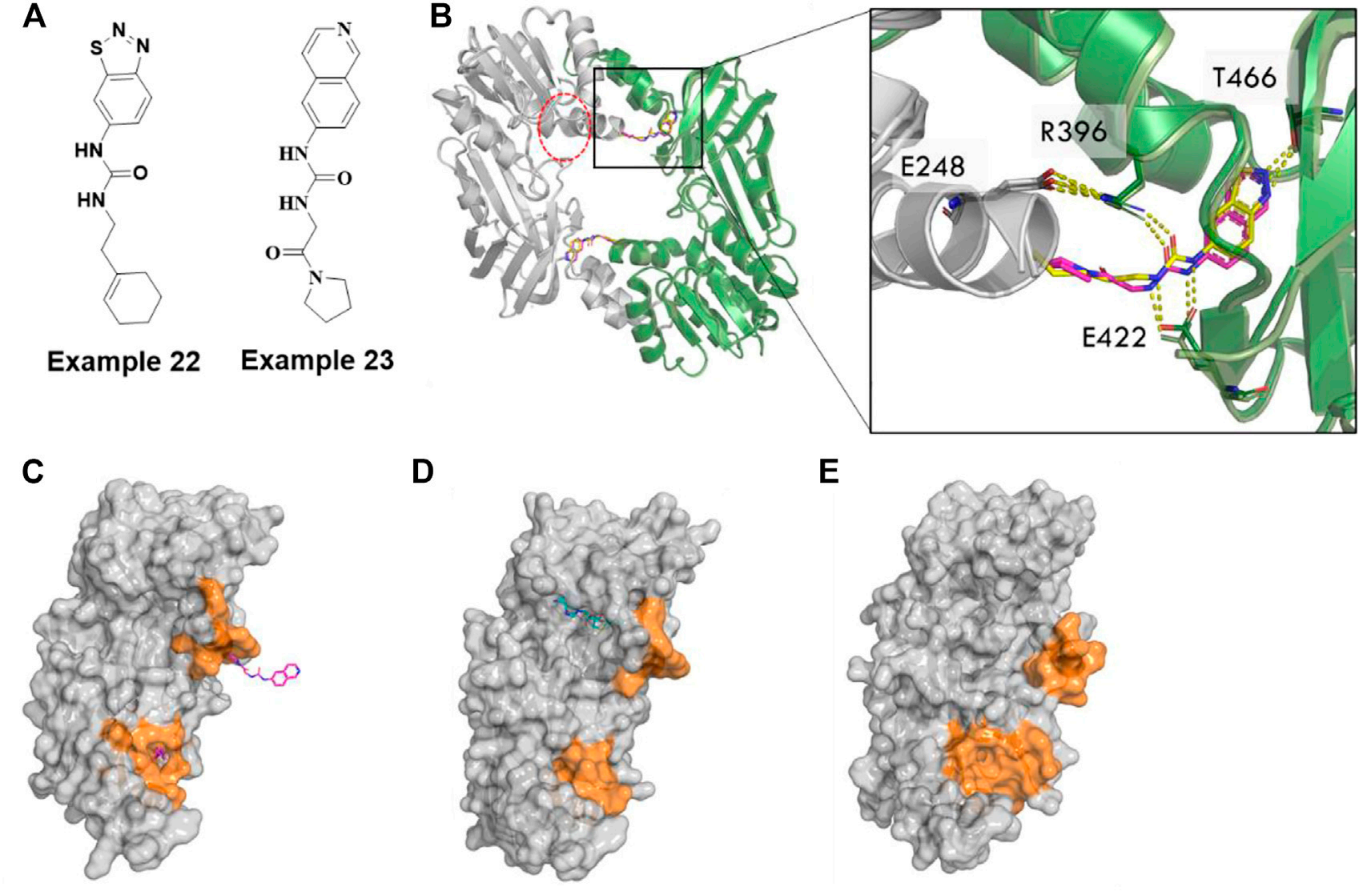
Allosteric inhibitors of PRMT3. **(A)** Chemical structures of allosteric inhibitors of PRMT3 **(B)** the inhibitor, example 22 (3SMQ, yellow sticks) overlayed with example 23(4RYL, pink sticks) binds a pocket at the base of the dimerization arm of one chain (green cartoon) and contacts the activation helix of the other monomer (gray cartoon), SAM binding site is circled in red dotted lines. **(C)** Monomer A chain of PRMT3 dimer (4RYL) is shown as gray surface with example 23 from both monomers represented as hotpink sticks. **(D)** PRMT1(1ORI) has cofactor in the position were allosteric pocket formed in PRMT3, whereas **(E)** apo PRMT4(3B3J) has a narrow cavity. Pocket forming region are colored by orange surface.

Although several potent small-molecule inhibitors targeting the SAM or substrate-competitive binding sites of PRMTs have been reported, it remains challenging to develop selective inhibitors due to the high homology of their binding sites (Hu et al., 2016). Recently, several novel allosteric inhibitors of PRMT3, 5, and 6 have been reported to have high selectivity across the PRMTs. Notably, the benzothiadiazole (example 22, Figure 10A) compound was the first identified allosteric inhibitor of PRMT3 by a Siarheyeva et al. (2012). The crystal structure of PRMT3 clearly illustrated that compound 2 (3SMQ, IC_50_ = 2.5 µM) binds at the dimer interface site which is distinct from both the SAM and substrate peptide binding pockets (Figure 10B) (Siarheyeva et al., 2012).

Kaniska et al. utilized this benzothiadiazole scaffold as a reference compound in a computational scaffold hopping along with docking. Ultimately, they selected 68 of 1,027 compounds for biochemical, biophysical, and cellular assays. The structure-based design protocol enabled them to discover a series of isoquinoline compounds, specifically example 23 (Figure 10A), that proved to be non-competitive inhibitors of PRMT3 (IC_50_ of .031 µM, K_d_ of .053 µM). These compounds proved to be selective against 31 other methytransferases and ∼250 non-epigenetic targets. They successfully solved the co-crystal structure of example 23 with PRMT3 (4RYL) (Kaniskan et al., 2015) which showed that it has essentially the same binding mode as the parent benzothiadiazole (example 22, Figure 10C). It occupies a site formed in the β-barrel of PRMT3, at the base of the dimerization arm, which is 15 Å far from the SAM binding site (Kaniskan et al., 2015).

Collective studies have suggested that the conserved α-helix across the class I PRMTs is critical for catalytic activity. The authors claimed that compound binding at the interface pocket induces a conformational stabilization at the N-terminus of the conserved α-helix by flipping the sidechain of R396 from its interaction with E422 out of the pocket, thereby preventing proper positioning of the substrate in a catalytically competent conformation (Figure 10B). Intriguingly, the corresponding location of the allosteric pocket of PRMT3 (4RYL, Figure 10C) to other type I PRMTs, for example, PRMT1 (1ORI) (Zhang and Cheng, 2003), has substrate bound nearby with small pockets (Figure 10D), whereas the apo CARM1/PRMT4 (3B3J) (Troffer-Charlier et al., 2007) has a narrow cavity which may form a deep pocket upon binding of small molecules (Figure 10E). Accordingly, it will be interesting to understand whether the analogous allosteric mechanism/pocket observed in PRMT3 also exists in other PRMTs.

**FIGURE 11:**
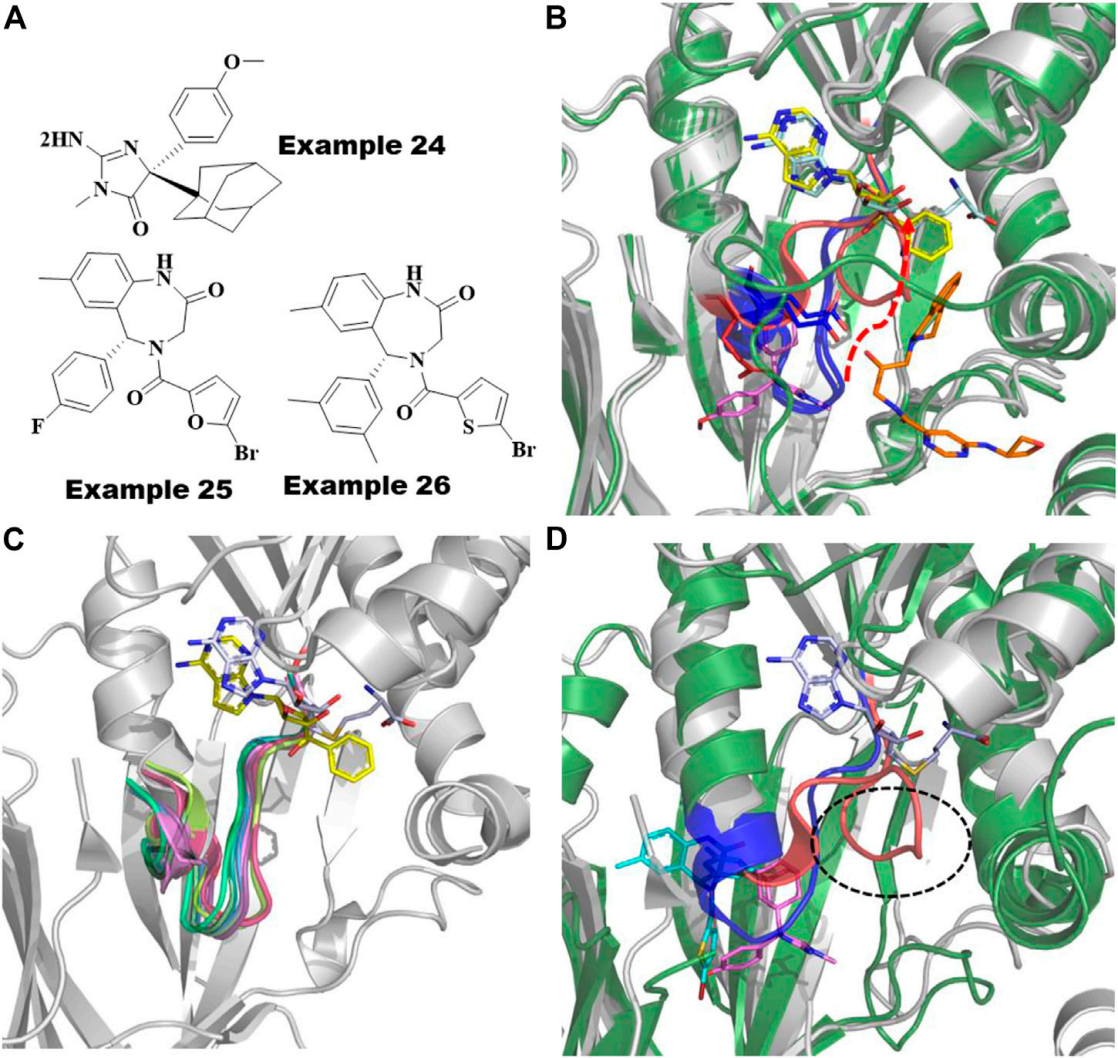
Allosteric inhibitors of PRMT5 and PRMT6. **(A)** Chemical structures of Example 24 known as BACE inhibitor as allosteric inhibitor of PRMT5; recently reported (R)-1 and (R)-2/SGC6870; Example 25 and 26 as allosteric inhibitors of PRMT6. **(B)** Overlayed crystal structures of PRMT5:MEP50-Compound 1 (pink sticks) with SAM-competitive inhibitor, EPZ015666 (yellow sticks, 4X60) and substrate competing inhibitor, LLY-283 (orange sticks, 6CKC). 12 amino acid loop that undergoes significant movement are highlighted as red cartoon for compound 1 bound complex and as blue cartoon for both SAM and substrate competing inhibitors. The movement of residue D444 involving polar interaction to allosteric inhibitor shown as red sticks and other conformation found in SAM or substrate competing inhibitors are shown as blue sticks. **(C)** Superposition of PRMTs, PRMT1 (1ORI, palegreen), PRMT2 (5JMQ, limegreen), PRMT3 (2YFT, teal), PRMT4 (5IH3, purple), PRMT5 (6CKC, red), PRMT6 (5EGS, smudge), PRMT7 (5EKU, warmpink), PRMT8 (5DST, marineblue), PRMT9 (6PDM, green) and PRMT10 (3ROQ, light magenta) highlighted the conserved structure of the 12-residue loop in the SAM/substrate bound conformation. **(D)** Overlayed Crystal structures of PRMT6-SGC6870/Example 26 (6W6D, green cartoon-cyan sticks) to PRMT5-Compound 1/Example 24 (6UXX, gray cartoon-magenta sticks). Allosteric pockets formed by flexible loop present in PRMT6 and PRMT5 are colored by blue and red, respectively and their diverse conformations are circled. SAM is shown as cyan sticks.

For PRMT5, the known BACE1/2 inhibitor, example 24 (Figure 11A), was first identified as an allosteric inhibitor by HTS and shown to have an EC_50_ of .016 µM in a biochemical methylation assay with a very slow on-rate of k_a_∼1,000 M^−1^ s^−1^ in binding kinetics (Palte et al., 2020; Xiong, 2021). Interestingly, the (S)-enantiomer was shown to be the more potent inhibitor of BACE1/2, whereas the (R)-enantiomer was shown to be ∼200-fold more potent for PRMT5. Moreover, the methylosome protein 50 (MEP50) has been shown to increase the enzymatic activity of PRMT5 (Ho et al., 2013). The co-crystal structure of the PRMT5:MEP50 in complex with the (R)-enantiomer of Compound 1 (6UXX) (Palte et al., 2020) shows a significant structural dislodgment in the backbone of the 12 amino acids loop (E435-L445) (Figure 11B). This loop forms a new binding pocket and blocks the SAM binding site thereby abrogating the substrate binding site (Figure 11B). A comparison of the crystal structure binding modes of the other PRMT5 inhibitors such as SAM-competitive and substrate-competitive inhibitors with this allosteric inhibitor revealed that significant movement only occurs in the loop while not affecting the remainder of the protein (Figure 11C). Although this amino acid loop has low sequence similarity across all the PRMTs, the structural alignment of backbone atoms indicates that it is positioned within <1.3 Å RMSD (Figure 11D) and is likely to follow the similar large loop movement to form an allosteric pocket in other PRMTs (Figure 11D).

Recently, Shen et al. reported the first highly selective and cell active allosteric inhibitor ((example 26, Figure 11A), IC_50_ = .077 µM) of PRMT6 by screening a diverse library of 5,000 compounds (Shen et al., 2021). For PRMT6, the R-enantiomer is the active isomer (IC50 = .39 µM) of example 26 (Figure 11A), whereas its S-enantiomer is inactive (IC_50_ > 100 μM). The cocrystal structures of PRMT6 with example 26 (6W6D) and example 25 (5WCF) revealed that the allosteric inhibitors are bound to a similar site as the allosteric pocket of PRMT5, described above, positioned inside the β-barrel domain and flexible loop (Figure 11A). As seen in PRMT5 (Figure 11C), the 12 amino acid loop also plays a major role in the formation of the allosteric pocket in PRMT6 (Figure 11D). The flexibility of this key loop, however, was not observed to block the SAM pocket for PRMT6. Taken together, it is promising to rationally exploit these dynamics to design selective allosteric inhibitors against other PRMTs.

## Discussion

As is evident from the PubMed analysis, over the last 2 decades (Figure 1), targeting allosteric sites for the discovery of novel therapeutic agents has gone from a curiosity to a mainstream effort. One of the primary benefits of targeting allosteric sites is that while a protein family invariably shares the same orthosteric site, they often have very different allosteric sites both in sequence and in structure. This is certainly the reason allosteric sites of protein kinases have been of the greatest interest. Across the protein kinase family, there are at least 12 different allosteric sites with a co-crystallized ligand (Figure 2), each of which is observed to be only functionally relevant for a small subset of the family (Laufkötter et al., 2022). This phenomenon also appears to be common for many protein families. Each of the families discussed here, kinases, tyrosine phosphatases, nuclear hormone receptors, proteases, and methyltransferases, have several known allosteric sites.

While traditional methods of structure-based design and virtual screening are relevant, there are several interesting and important computational challenges unique to understanding and modeling allostery. The first challenge is understanding why an allosteric site that has proven fruitful for one target cannot be exploited for other family members. This is particularly apparent with the MEK/WNK example discussed in the protein kinase section. Identifying additional protein kinases where the type III allosteric site is accessible and able to impede the kinase activity is clearly of great interest. The challenge is also apparent with RORg and the nuclear hormone receptor family. Despite significant efforts in developing drugs across the family, RORg is the only family member for which a potent antagonist that binds in the helix 12 site has been found.

A second important challenge is to distinguish sites that allow for only modest small molecule binding affinity (e.g., >1 µM) form those that lead to drug-like affinity. This challenge is exemplified with the effort on the PTP1b allosteric site near α7. Despite significant effort, no inhibitor at this site has broken the 1 µM barrier. In cases such as this, identifying when and where deeper sub-pockets can be induced will be critical. A related challenge is to identify cryptic pockets that are not at all apparent without a bound ligand.

A third important challenge unique to allosteric sites is identifying those sites that are functionally relevant. Several methods, such as molecular dynamics, normal mode analysis and elastic network models, have been developed to understand the conformational coupling between two sites. The PTP1B and RORg examples demonstrate that some progress has been made in this area. As more examples emerge, new tools are developed and large independent assessments are made of available methods, they may become more commonly used prospectively.

Not all allosteric sites, however, work *via* conformational coupling with the orthosteric site. For example, allosteric sites can be functionally relevant because they block a necessary scaffolding interaction as discussed in cathepsin K or preventing a post-translational modification. Thus, a fourth challenge is to identify these types of sites. Identifying these sites will necessarily involve a larger view of the protein in the context of its physiological environment.

Addressing these problems computationally will be challenging. To fully do so will require a view beyond the 3D structure of an isolated target of interest. Fortunately, there is a vast amount of publicly available information to address these problems. This data includes sequence data from hundreds of genomes, disease relevant mutational data derived by comparing diseased to normal tissues, data on cell wide post translational modifications under a variety of stimulations, hundreds of thousands of crystal structures of proteins, and large easily accessible datasets of small molecule modulators for many protein families. Only by integrating all this data can we hope to develop the holistic view of a protein and its family to select the best strategy to target a protein allosterically, the best molecules to interrogate the allosteric site and the best assays to identify the right modulators.
